# Association between the Telomerase Reverse Transcriptase (*TERT*) rs2736098 Polymorphism and Cancer Risk: Evidence from a Case-Control Study of Non-Small-Cell Lung Cancer and a Meta-Analysis

**DOI:** 10.1371/journal.pone.0076372

**Published:** 2013-11-19

**Authors:** Haijian Wu, Naian Qiao, Yang Wang, Man Jiang, Shikun Wang, Cuihong Wang, Likuan Hu

**Affiliations:** 1 Department of Radiation Oncology, Qilu Hospital Affiliated to Shandong University, Jinan, Shandong Province, China; 2 Department of Gastroenterology, Provincial Hospital Affiliated to Shandong University, Jinan, Shandong Province, China; Cedars Sinai Medical Center, United States of America

## Abstract

**Background:**

A common genetic variant, telomerase reverse transcriptase (*TERT*) rs2736098, was recently reported to be associated with lung cancer risk in Caucasians. In addition, many studies have investigated the role of this polymorphism in the etiology of cancer of various organs. Nevertheless, the results of related case-control studies remain inconsistent.

**Methods:**

We hypothesized that the genetic risk variant identified in Caucasians may potentially influence the susceptibility to lung cancer in the Chinese population. To test this hypothesis, a case-control study including 539 non-small-cell lung cancer (NSCLC) cases and 627 cancer-free controls was conducted. Furthermore, to investigate the association between rs2736098 and cancer risk, a meta-analysis based on previously published studies and our case-control study was also performed.

**Results:**

Multivariate logistic regression demonstrated that individuals carrying the A allele or the AA genotype exhibited a significantly elevated risk of NSCLC compared with those carrying the G allele or GG genotype (A vs. G: OR = 1.21, 95% CI = 1.02–1.43, *P* = 0.028; AA vs. GG: OR = 1.48, 95% CI = 1.05–2.09, *P* = 0.025). Additionally, this association was stronger among adenocarcinoma cases (AA vs. GG: OR = 1.67, 95% CI = 1.12–2.50, *P* = 0.013; A vs. G: OR = 1.28, 95% CI = 1.05–1.57, *P* = 0.016). In the meta-analysis, a borderline significant association between the rs2736098 polymorphism and overall cancer risk was observed (AA vs. GG: OR = 1.25, 95% CI = 1.07–1.46; AA vs. AG+GG: OR = 1.22, 95% CI = 1.06–1.41; additive model: OR = 1.10, 95% CI = 1.02–1.18), and further stratifications demonstrated a moderately increased risk for lung and bladder cancer, Asian ethnicity and hospital-based studies.

**Conclusions:**

Our results suggest that the rs2736098 polymorphism may contribute to the risk of lung cancer, especially adenocarcinoma, in the Chinese population. In addition, the current meta-analysis indicates that this genetic variant is only weakly associated with overall cancer risk. However, the rs2736098 polymorphism may affect individual susceptibility to lung and bladder cancer. Further studies are needed to validate our findings.

## Introduction

Worldwide, lung cancer was the leading cause of cancer deaths in males and the second leading cause of cancer deaths in females in 2008. The geographical and temporal patterns of lung cancer incidence are largely determined by tobacco consumption. Lung cancer rates are increasing in countries such as China and several other countries in Asia and Africa, where the smoking prevalence continues to either increase or show signs of stability [1]. Approximately 80% of the 1.3 billion current smokers worldwide live in low- and middle-income countries, with over 300 million in China alone [2]. Non-small-cell lung cancer (NSCLC), which includes two main histological types, squamous cell carcinoma (SQC) and adenocarcinoma (ADC), accounts for nearly 85% of all lung cancer cases. Despite considerable therapeutic progress, the prognosis of NSCLC patients remains poor [3].

The development of lung cancer appears to be the result of a complex interaction between environmental exposures and genetic factors. Recently, independent genome-wide association studies (GWAS) [4]–[9] have demonstrated that single nucleotide polymorphisms (SNPs) in three separate chromosomal regions (5p15, 6p21, and 15q25), which contain genes that regulate nicotinic acetylcholine receptor (nAChR) and telomerase production, are significantly associated with lung cancer risk. 5p15.33, a crucial genomic region for telomere biology, contains two well-known genes: telomerase reverse transcriptase (*TERT*) and cleft lip and palate trans-membrane 1-like (*CLPTM1L*). TERT protein is the telomerase catalytic subunit that elongates telomeres and serves as a key regulator of telomerase activity. Telomeres, consisting of TTAGGG repeats that undergo shortening with each cell replication cycle, have long been known to be essential for the preservation of chromosomal integrity [10]. As telomerase and the control of telomere length are intimately linked to the development of many tumor types, scientific attention has focused on the possibility of targeting telomerase and telomere-binding proteins in therapeutic strategies against cancer [11], [12]. Recently, it has been reported that genetic variants at the 5p15.33 locus, which contains the *TERT* gene (encoding the catalytic subunit of telomerase), are involved in the susceptibility of many tumor types [13], [14].

A common genetic variant, *TERT* rs2736098, which is located on chromosome 5p15.33, was recently identified as a susceptibility locus for lung cancer in a combined analysis of Icelandic and European sample sets [15]. More recently, a Korean population study of 720 lung cancer patients and 720 healthy controls revealed that the *TERT* A variant genotype is associated with a significantly increased risk of lung cancer [16]. Given the relevance of this genomic region (5p15.33) to tumor biology and the need to verify these associations in diverse populations with different ancestries, we hypothesized that the risk genetic variant (rs2736098) identified by previous studies of Caucasian and Korean populations may potentially influence the susceptibility to lung cancer in the Chinese Han population. To test this hypothesis, we genotyped the SNP rs2736098 and analyzed its association with the risk of lung cancer in a case–control study of 539 NSCLC cases and 627 cancer-free controls matched by age and gender in a Chinese Han population.

Furthermore, many studies have investigated the role of this polymorphism in the etiology of cancer of various organs, including the bladder, liver, and breast [17]–[27]. However, the results of related published case-control studies remain conflicting rather than conclusive. Therefore, to further explore the association between the *TERT* rs2736098 polymorphism and cancer risk, a meta-analysis based on previously published studies and our case-control study was also performed.

## Materials and Methods

### Case-control study

#### Study population

To exclude the possible effects of ethnicity, all subjects in this study were genetically unrelated ethnic Han Chinese. The cases included 539 newly diagnosed NSCLC patients who were admitted to the Qilu Hospital of Shandong University (Jinan, China) between 2010 and 2012. Of these NSCLC patients, 293 patients had adenocarcinomas (ADC) and 246 had squamous cell carcinomas (SQC). Meanwhile, 627 cancer-free controls were selected from the same hospital and were frequency-matched to cases by age and sex. Subjects who were relatives or had histories of malignancy and other major diseases were excluded from this study. In addition, a structured questionnaire was completed for each case and control by a trained interviewer to collect demographic data and other relevant information, including age, sex, and smoking status. Those individuals who smoked <1 cigarette per day and for <1 year were defined as nonsmokers; otherwise, the patients were considered smokers. All participants were given an explanation of the study, and written informed consent was obtained from each participant. This study was conducted under the approval of the Ethics Committees of Qilu Hospital affiliated to Shandong University.

#### DNA extraction and SNP genotyping

Blood samples were collected from all participants at the time of recruitment. Genomic DNA was extracted from peripheral blood obtained from each participant using the DNA Extraction Kit (Tiangen Biotech (Beijing) Co., Ltd.) according to the manufacturer's protocol. The *TERT* SNP rs2736098 was genotyped using the TaqMan methodology in 96-well plates and read with the Sequence Detection Software (SDS, version 1.4) on an Applied Biosystems (ABI) 7500 Real-Time PCR System.

#### Statistical analysis

The Pearson χ^2^ test was employed to evaluate the differences in the distributions of selected characteristics between the cases and controls. The goodness-of-fit χ^2^ test was adopted to assess Hardy-Weinberg equilibrium (HWE) in the controls. Multivariate logistic regression analysis was used to estimate odds ratios (ORs) and their 95% confidence intervals (CIs) for the effect of rs2736098 polymorphism on NSCLC risk. In addition, stratified analyses by histological types were further performed to evaluate the role of rs2736098 in NSCLC risk. All statistical tests were two-sided, and statistical significance was accepted as *P*<0.05.

### Meta-analysis

#### Identification and eligibility of relevant studies

To further investigate the association between the *TERT* rs2736098 polymorphism and cancer risk, a meta-analysis based on previously published studies and our case-control study was performed. We searched the PubMed and ISI Web of Science databases for all articles on the association between the *TERT* rs2736098 polymorphism and cancer risk (last search update 5th June 2013). The following search terms were used in isolation and in combination with one another: “telomerase reverse transcriptase or *TERT* or 5p15.33”, “polymorphism or variant or variation”, and “cancer or carcinoma or tumor”. The search was limited to English language papers and human studies. In addition, we screened the reference lists for all included studies, reviews and meta-analyses. When multiple publications reported on the same or overlapping data, we selected the most recent publication with the most subjects. Studies included in our meta-analysis had to meet the following inclusion criteria: (1) evaluation of the *TERT* rs2736098 polymorphism and cancer risk; (2) a case-control design; (3) sufficient genotype data for the calculation of odds ratios (OR) with 95% confidence intervals (CIs); and (4) written in English. The major reasons for exclusion of studies included (1) the lack of a control group; (2) duplicates of previous publications; (3) reviews, comments or editorials; and (4) a lack of usable data on genotype frequencies.

#### Data extraction

Two investigators (Wu H and Wang Y) extracted information from all eligible publications independently according to the inclusion criteria listed above. Disagreements were resolved by discussion until consensus was achieved on every item. In the present study, the following characteristics were collected: the first author's last name, the year of publication, the country of origin, ethnicity, cancer type, the source of the control groups (population- or hospital-based controls), the genotyping method, and the frequencies of genotypes in cases and controls. For studies including subjects of different ethnic groups, genotype frequencies and other information were extracted separately for each ethnic group whenever possible [21].

#### Statistical analysis

We first assessed the Hardy-Weinberg equilibrium (HWE) for the controls in each study. The strength of the association between the *TERT* rs2736098 polymorphism and cancer risk was evaluated by the odds ratios (ORs) with 95% confidence intervals (CIs). The pooled ORs were calculated for homozygote comparison (AA vs. GG), heterozygote comparison (AG vs. GG), the dominant genetic model (AA+AG vs. GG), the recessive genetic model (AA vs. AG+GG), and the additive genetic model (2*AA+AG vs. 2*GG+AG). Stratified analyses were performed by cancer type (if one cancer type contained less than two individual studies, it was combined into the ‘other cancers’ group), ethnicity and source of the controls. The evaluation of the meta-analysis results included an examination of the heterogeneity, an analysis of the sensitivity, and an examination for publication bias. Heterogeneity was checked by the chi-square-based Q-test [28]. If the result of this heterogeneity test was *P*<0.05, then the pooled ORs were calculated using the random effects model (the DerSimonian and Laird method) [29]. Otherwise, if the result of this heterogeneity test was *P*>0.05, the fixed-effects model was selected (the Mantel–Haenszel method) [30]. We also used the *I^2^* statistic to efficiently test for heterogeneity, with *I^2^*<25%, 25–75% and >75% representing low, moderate and high degrees of inconsistency, respectively [31], [32]. Additionally, sensitivity analyses were performed by omitting each study to reflect the influence of the individual data on the summary ORs. Finally, literature publication bias was estimated using the Begg's funnel plot and Egger's test (*P*<0.05 was considered a significant publication bias) [33], [34]. All statistical analyses were performed using the STATA software (version 12.0; Stata Corporation, College Station, TX).

## Results

### Results of the case-control study

#### Population characteristics

The characteristics of the cases and controls are presented in Table 1. A total of 539 NSCLC cases and 627 cancer-free controls were enrolled in this study. There were no significant differences in the distributions of sex (*P* = 0.403) and age (*P* = 0.688) between the case and control groups. Males represented 79.7% of the control group and 77.7% of the case group. Of the 539 NSCLC cases, 293 (54.4%) were adenocarcinomas, and 246 (45.6%) were squamous cell carcinomas. Approximately 51.8% of cases were smokers, compared with 43.1% of controls (*P* = 0.003).

**Table 1:** Selected characteristics of non-small-cell lung cancer cases and controls.

Characteristics	N (%)		*P* *
	Cases (n = 539)	Controls (n = 627)	
Age (years)			
≤60	278(51.6)	316(50.4)	0.688
>60	261(48.4)	311(49.6)	
Sex			
Male	419(77.7)	500(79.7)	0.403
Female	120(22.3)	127(20.3)	
Smoking status			
Ever	279(51.8)	270(43.1)	0.003
Never	260(48.2)	357(56.9)	
Histology			
SQC	246(45.6)		
ADC	293(54.4)		

Abbreviations: ADC, adenocarcinoma; SQC, squamous cell carcinoma.

* *P* value was calculated by the χ^2^ test.

#### Association between the *TERT* rs2736098 polymorphism and NSCLC risk

Data for the genotype frequencies and the association between the *TERT* rs2736098 polymorphism and NSCLC risk are shown in Table 2. The distribution of genotypes among the control subjects was in accordance with Hardy–Weinberg equilibrium (*P* = 0.361). The multivariate logistic regression model demonstrated that individuals carrying the A allele or AA genotype exhibited a significantly elevated risk of NSCLC compared with those carrying the G allele or GG genotype, after adjusting for age, gender and smoking status (A vs. G: OR = 1.21, 95% CI = 1.02–1.43, *P* = 0.028; AA vs. GG: OR = 1.48, 95% CI = 1.05–2.09, *P* = 0.025).

**Table 2:** Association between the rs2736098 polymorphism and non-small-cell lung cancer risk in a Chinese Han population.

Genotypes	Cases (n = 539), N (%)	Controls^a^ (n = 627), N (%)	OR (95%CI)^b^	*P* b
Total				
GG	205 (38.0)	263(41.9)	1.00	
AG	232(43.0)	278(44.3)	1.09(0.85–1.41)	0.501
AA	102(18.9)	86 (13.7)	1.48(1.05–2.09)	0.025
AA+AG	334/205	364/263	1.18(0.93–1.50)	0.163
A allele			1.21(1.02–1.43)	0.028
ADC				
GG	106(36.2)	263(41.9)	1.00	
AG	126(43.0)	278(44.3)	1.13(0.83–1.54)	0.450
AA	61(20.8)	86(13.7)	1.67(1.12–2.50)	0.013
AA+AG	187/106	364/263	1.25(0.94–1.67)	0.132
A allele			1.28(1.05–1.57)	0.016
SQC				
GG	99(40.2)	263(41.9)	1.00	
AG	106(43.1)	278(44.3)	1.07(0.77–1.49)	0.672
AA	41(16.7)	86 (13.7)	1.23(0.78–1.94)	0.375
AA+AG	147/99	364/263	1.12(0.82–1.52)	0.487
A allele			1.11(0.89–1.38)	0.363

Abbreviations: OR, odds ratio; CI, confidence interval; ADC, adenocarcinoma; SQC, squamous cell carcinoma.

a The observed genotype frequency among the control subjects was in agreement with the Hardy–Weinberg equilibrium (*P* = 0.361).

b ORs and their corresponding 95% CIs were calculated by multivariate logistic regression after adjusting for age, sex and smoking status.

The association between the *TERT* rs2736098 polymorphism and NSCLC risk was further examined by stratifying the subjects according to tumor histology. When analyzed according to the histological type, the effect of the *TERT* rs2736098 polymorphism on the NSCLC risk was significant for adenocarcinomas (A vs. G: OR = 1.28, 95% CI = 1.05–1.57, *P* = 0.016; AA vs. GG: OR = 1.67, 95% CI = 1.12–2.50, *P* = 0.013), but not for squamous cell carcinomas (A vs. G: OR = 1.11, 95% CI = 0.89–1.38, *P* = 0.363; AA vs. GG: OR = 1.23, 95% CI = 0.78–1.94, *P* = 0.375; AA+AG vs. GG: OR = 1.12, 95% CI = 0.82–1.52, *P* = 0.487) ( Table 2).

### Results of the meta-analysis

#### Study characteristics

Figure S1 presents the literature search and study selection procedures. Eleven articles [16]–[26] on 12 case-control studies plus the present study, encompassing a total of 10,044 cancer cases and 12,480 controls, were finally included in this meta-analysis. These 13 studies included 3 lung cancer studies, 2 bladder cancer studies, 2 hepatocellular carcinoma (HCC) studies, and 6 other cancer studies (including breast cancer and cervical cancer, among others). There were 5 population-based studies and 8 hospital-based studies. Four studies were conducted in European descendants, and 9 studies were conducted in Asian descendants. The genotype distributions in the controls of all studies were in agreement with HWE, with the exception of 2 studies (*P*<0.05) [21], [24], which were further tested in the sensitivity analyses. Table 3 presents the characteristics of the included studies.

**Table 3:** Characteristics of the studies included in the meta-analysis.

First author	Published year	Country	Ethnicity	Cancer type	Control source	Genotyping method	Cases			Controls			*P* of HWE
							AA	AG	GG	AA	AG	GG	
Savage [17]	2007	Poland	Caucasian	Breast cancer	PB	TaqMan	97	699	1171	141	811	1313	0.294
Choi [16]	2009	Korea	Asian	Lung cancer	HB	PCR	87	322	311	55	320	345	0.102
Liu [18]	2010	USA	Caucasian	SCCHN	HB	TaqMan	72	419	588	78	461	576	0.271
Chen [19]	2011	China	Asian	Glioma	HB	PCR	141	461	351	117	486	430	0.246
Ding [20]	2011	China	Asian	HCC	HB	TaqMan	210	563	500	198	604	526	0.255
Gago-Dominguez [21]	2011	USA	Caucasian	Bladder cancer	PB	TaqMan	43	189	217	43	210	278	0.706
Gago-Dominguez [21]	2011	China	Asian	Bladder cancer	PB	TaqMan	85	236	178	54	270	203	0.009
Wang [22]	2012	China	Asian	Cervical cancer	PB	TaqMan	174	444	375	138	480	397	0.710
Hofer [23]	2012	Austria	Caucasian	Colorectal cancer	PB	TaqMan	6	45	86	119	623	963	0.186
Zhang [24]	2013	China	Asian	HCC	HB	PCR	61	206	133	65	158	177	0.004
Li [25]	2013	China	Asian	Lung cancer	HB	TaqMan	88	207	173	67	250	227	0.886
Sheng [26]	2013	China	Asian	ALL	HB	TaqMan	93	238	236	96	298	276	0.286
Present study	2013	China	Asian	Lung cancer	HB	TaqMan	102	232	205	86	278	263	0.361

Abbreviations: SCCHN, squamous cell carcinoma of the head and neck; HCC, hepatocellular carcinoma; ALL, acute lymphoblastic leukemia; PB, population based; HB, hospital based; HWE, Hardy-Weinberg equilibrium.

#### Main meta-analysis results

**Figure 1:**
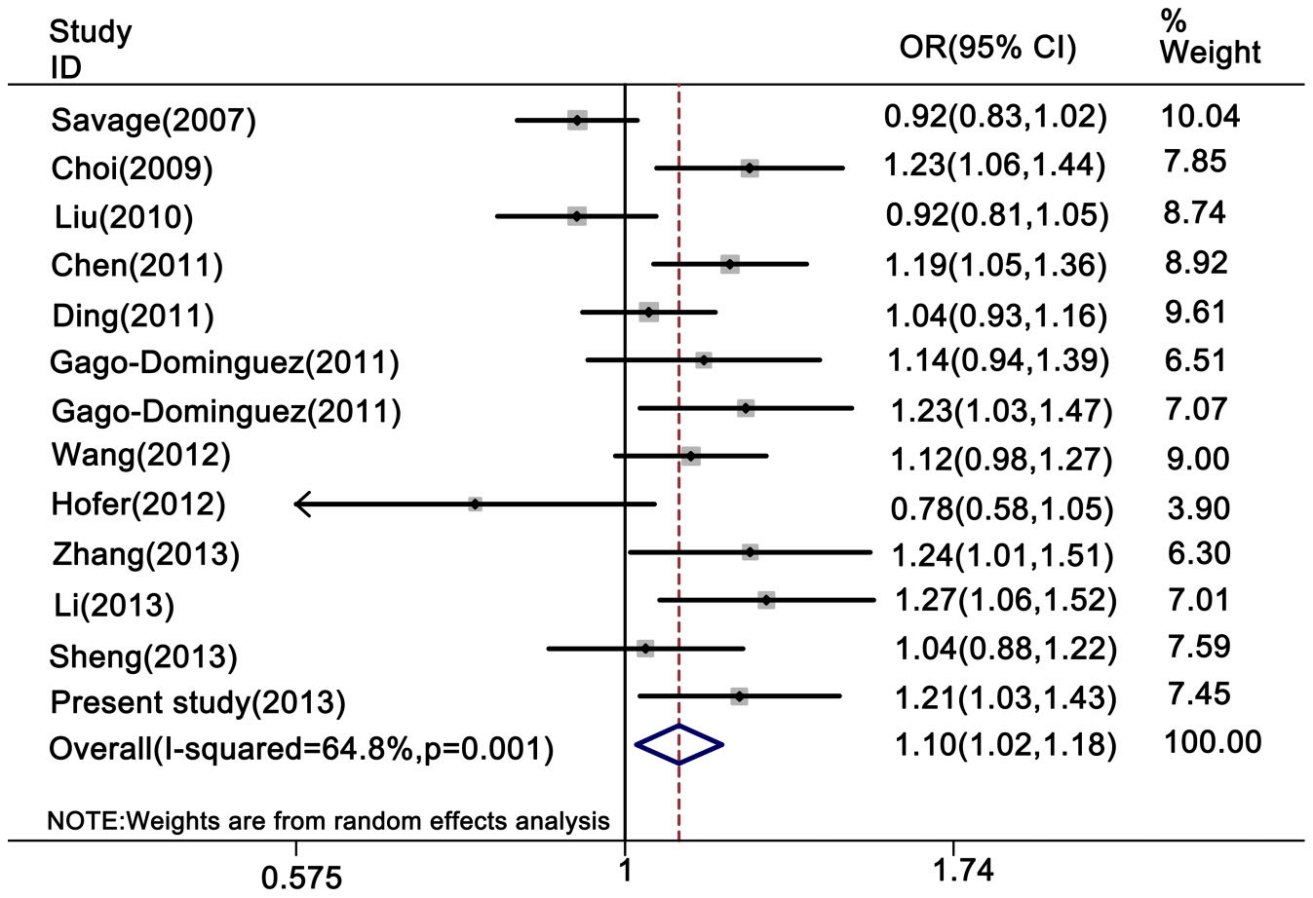
Forest plot of cancer risk associated with the rs2736098 polymorphism (additive model). The squares and horizontal lines correspond to the study-specific OR and 95% CI. The area of the squares reflects the study-specific weight (inverse of the variance). The diamonds represent the summary OR and 95% CI. The rs2736098 polymorphism was weakly associated with an increased risk of cancer in the additive model.

**Figure 2:**
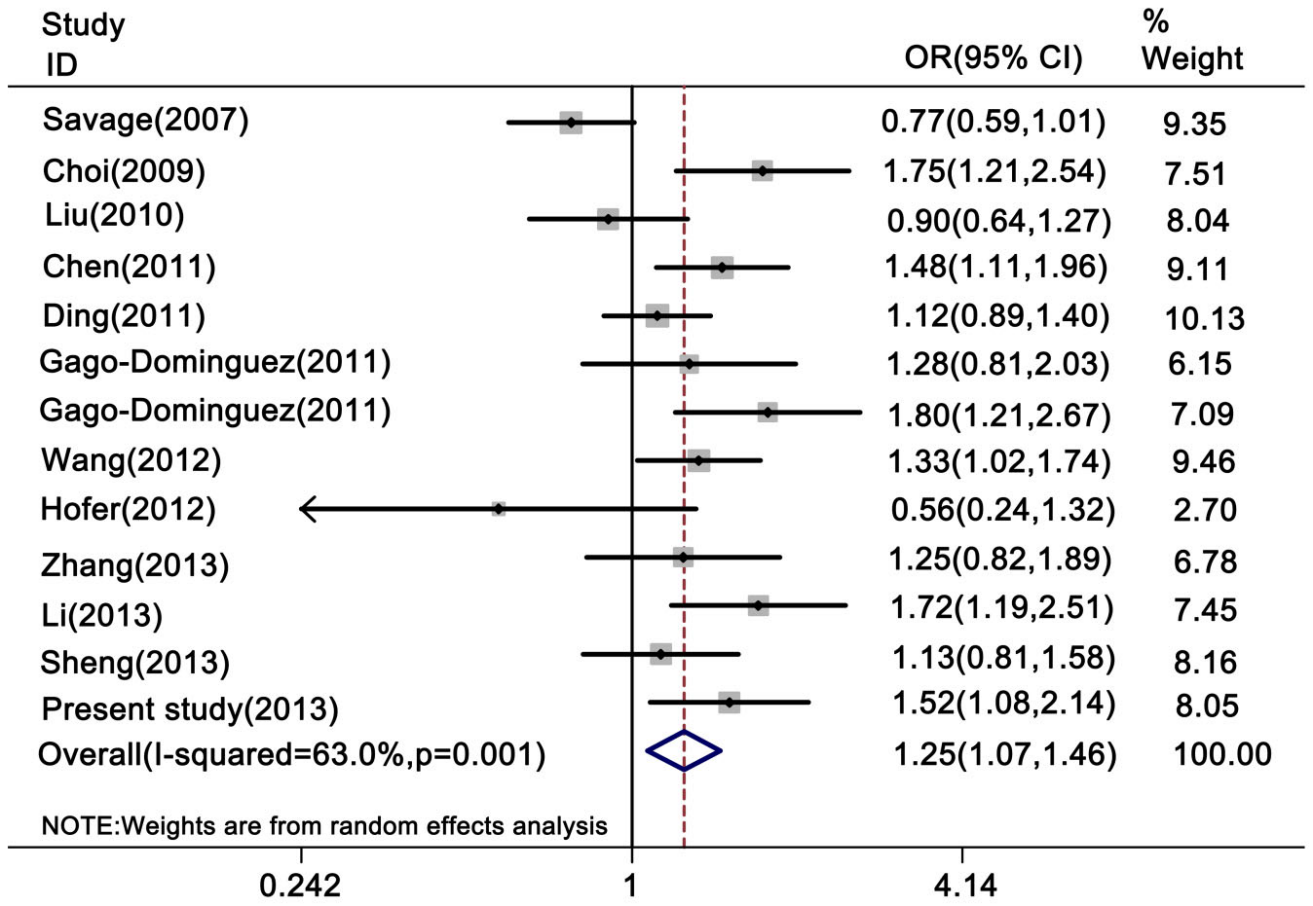
Forest plot of cancer risk associated with the rs2736098 polymorphism (AA vs. GG). The rs2736098 polymorphism was associated with an increased risk of cancer in the homozygote comparison (AA vs. GG).

Overall, as shown in Table 4, a borderline significant association was observed between the *TERT* rs2736098 polymorphism and overall cancer risk in the homozygote comparison (AA vs. GG: OR = 1.25, 95% CI = 1.07–1.46), recessive genetic model (AA vs. AG+GG: OR = 1.22, 95% CI = 1.06–1.41) and additive genetic model (2*AA+AG vs. 2*GG+AG: OR = 1.10, 95% CI = 1.02–1.18) (Figure 1 and Figure 2), but no statistically significant association was found in the heterozygote comparison (AG vs. GG: OR = 1.02, 95% CI = 0.97–1.08) or the dominant genetic model (AA+AG vs. GG: OR = 1.08, 95% CI = 0.99–1.18).

**Table 4:** Meta-analysis of the rs2736098 polymorphism in association with cancer risk.

Variables	N	AA vs. GG			AG vs. GG			Dominant			Recessive			Additive		
								(AA+AG vs. GG)			(AA vs. AG+GG)			(2*AA+AG vs. 2*GG+AG)		
		OR(95%CI)	*P_het_*	*I^2^*	OR(95%CI)	*P_het_*	*I^2^*	OR(95%CI)	*P_het_*	*I^2^*	OR(95%CI)	*P_het_*	*I^2^*	OR(95%CI)	*P_het_*	*I^2^*
Total	13	**1.25(1.07–1.46)**	0.001	63.0	1.02(0.97–1.08)	0.056	41.8	1.08(0.99–1.18)	0.010	54.3	**1.22(1.06–1.41)**	0.002	61.3	**1.10(1.02–1.18)**	0.001	64.8
Cancer type																
Lung cancer	3	**1.65(1.34–2.04)**	0.829	0.0	1.09(0.95–1.26)	0.969	0.0	**1.20(1.05–1.37)**	0.976	0.0	**1.58(1.30–1.92)**	0.841	0.0	**1.24(1.12–1.36)**	0.936	0.0
HCC	2	1.15(0.94–1.40)	0.642	0.0	1.12(0.97–1.30)	0.001	90.3	1.25(0.80–1.94)	0.007	86.4	1.08(0.89–1.30)	0.379	0.0	1.11(0.94–1.31)	0.147	52.5
Bladder cancer	2	**1.55(1.11–2.15)**	0.275	16.1	1.07(0.89–1.29)	0.448	0.0	1.15(0.96–1.38)	0.831	0.0	**1.50(1.01–2.22)**	0.169	47.1	**1.19(1.04–1.35)**	0.598	0.0
Other cancers	6	1.05(0.82–1.35)	0.006	69.7	0.97(0.90–1.05)	0.369	7.4	0.99(0.89–1.10)	0.078	49.5	1.07(0.86–1.33)	0.013	65.2	1.01(0.90–1.12)	0.005	70.0
Ethnicity																
Caucasian	4	0.88(0.69–1.13)	0.208	34.0	0.95(0.87–1.05)	0.341	10.4	0.94(0.83–1.06)	0.205	34.5	0.89(0.72–1.10)	0.307	16.9	0.95(0.85–1.06)	0.136	45.8
Asian	9	**1.39(1.23–1.57)**	0.257	20.9	1.07(0.99–1.15)	0.087	42.1	**1.14(1.05–1.24)**	0.207	26.7	**1.34(1.19–1.52)**	0.132	35.8	**1.15(1.09–1.21)**	0.394	4.9
Source of control																
PB	5	1.12(0.78–1.61)	0.002	76.9	0.98(0.90–1.07)	0.642	0.0	1.01(0.90–1.13)	0.203	32.8	1.13(0.80–1.60)	0.001	77.5	1.04(0.91–1.19)	0.007	71.8
HB	8	**1.31(1.12–1.54)**	0.071	46.4	1.05(0.98–1.14)	0.019	58.3	**1.13(1.00–1.26)**	0.015	59.9	**1.26(1.09–1.45)**	0.088	43.6	**1.13(1.04–1.22)**	0.020	57.8
Publication bias																
Begg's test		*P* = 0.583			*P* = 0.246			*P* = 0.200			*P* = 0.855			*P* = 0.300		
Egger's test		*P* = 0.795			*P* = 0.220			*P* = 0.123			*P* = 0.913			*P* = 0.290		

*P_het_*: test for heterogeneity; OR: odds ratio; CI: confidence interval; N: number of comparisons.

The figures given in bold indicate statistically significant values.

In the subgroup analysis according to cancer type, significantly increased risk was observed in lung cancer (AA vs. GG: OR = 1.65, 95% CI = 1.34–2.04; dominant model: OR = 1.20, 95% CI = 1.05–1.37; recessive model: OR = 1.58, 95% CI = 1.30–1.92; additive model: OR = 1.24, 95% CI = 1.12–1.36) and bladder cancer (AA vs. GG: OR = 1.55, 95% CI = 1.11–2.15; recessive model: OR = 1.50, 95% CI = 1.01–2.22; additive model: OR = 1.19, 95% CI = 1.04–1.35). However, no evidence of association was observed in any genetic model between the *TERT* rs2736098 polymorphism and the risk of HCC or other cancers. When stratified by ethnicity, significantly increased risk was observed in the Asian population (AA vs.GG: OR = 1.39, 95% CI = 1.23–1.57; dominant model: OR = 1.14, 95% CI = 1.05–1.24; recessive model: OR = 1.34, 95% CI = 1.19–1.52; additive model: OR = 1.15, 95% CI = 1.09–1.21) in all genetic models tested, with the exception of the heterozygote comparison (AG vs. GG: OR = 1.07, 95% CI = 0.99–1.15). Nevertheless, no significant association was observed in the European population. In the subgroup analysis by the source of controls, significantly increased risk was observed in hospital-based studies (AA vs. GG: OR = 1.31, 95% CI = 1.12–1.54; dominant model: OR = 1.13, 95% CI = 1.00–1.26; recessive model: OR = 1.26, 95% CI = 1.09–1.45; additive model: OR = 1.13, 95% CI = 1.04–1.22) but was not observed in population-based studies. The main results of this meta-analysis and the heterogeneity test are presented in Table 4.

#### Test of heterogeneity

For the overall comparisons, significant heterogeneity was observed in four genetic models (AA vs. GG: *P_het_*_._ = 0.001, *I* ^2^ = 63.0%; AA+AG vs. GG: *P_het._* = 0.010, *I* ^2^ = 54.3%; AA vs. AG+GG: *P_het_*_._ = 0.002, *I* ^2^ = 61.3%; 2*AA+AG vs. 2*GG+AG: *P_het_*_._ = 0.001, *I* ^2^ = 64.8%). However, the heterogeneity decreased markedly after stratification, especially in the subgroups of lung cancer (AA vs.GG: *P_het_*_._ = 0.829, *I* ^2^ = 0.0%; AG vs.GG: *P_het_*_._ = 0.969, *I* ^2^ = 0.0%; dominant model: *P_het_*_._ = 0.976, *I* ^2^ = 0.0%; recessive model: *P_het._* = 0.841, *I* ^2^ = 0.0%; additive model: *P_het_*_._ = 0.936, *I* ^2^ = 0.0%) and bladder cancer (AA vs.GG: *P_het._* = 0.275, *I* ^2^ = 16.1%; AG vs.GG: *P_het_*_._ = 0.448, *I* ^2^ = 0.0%; dominant model: *P_het_*_._ = 0.831, *I* ^2^ = 0.0%; recessive model: *P_het_*_._ = 0.169, *I* ^2^ = 47.1%; additive model: *P_het._* = 0.598, *I* ^2^ = 0.0%). When stratified by ethnicity, heterogeneity was not observed in the subgroups of Asian and European populations (*P_het._*>0.05 in all genetic comparisons) (Table 4).

#### Sensitivity analysis

**Figure 3:**
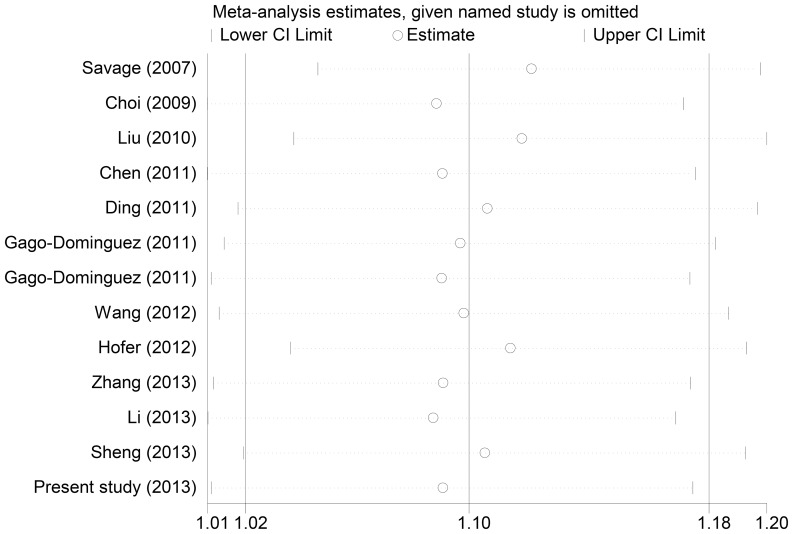
Sensitivity analysis of the summary OR on the association between the rs2736098 polymorphism and cancer risk under the additive model. The results were computed by omitting each study (left column) in turn. Meta-analysis random-effect estimates were used. Bars, 95% CI.

In the sensitivity analyses, the influence of each study on the pooled OR was checked individually by repeating the meta-analysis while omitting each study. Although the genotype distributions of the control groups in the studies by Gago-Dominguez et al. [21] and Zhang et al. [24] did not follow Hardy–Weinberg equilibrium, the corresponding pooled OR and between-study heterogeneity were not significant altered with or without these two studies. Sensitivity analyses indicated that the two independent studies by Savage et al. [17] and Liu et al. [18] were the main origin of the heterogeneity in the overall comparisons (Figure 3). The heterogeneity was effectively decreased or removed after exclusion of these two studies (AA vs. GG: OR = 1.35, 95%CI = 1.22–1.50, *P_het_*_._ = 0.158, *I* ^2^ = 30.3%; dominant model: OR = 1.12, 95%CI = 1.05–1.20, *P_het_*_._ = 0.119, *I* ^2^ = 35.0%; additive model: OR = 1.14, 95%CI = 1.09–1.19, *P_het_*_._ = 0.142, *I* ^2^ = 32.1%). Furthermore, none of the pooled ORs was significantly affected by any single study, suggesting that the results of this meta-analysis were relatively stable.

#### Publication bias assessment

**Figure 4:**
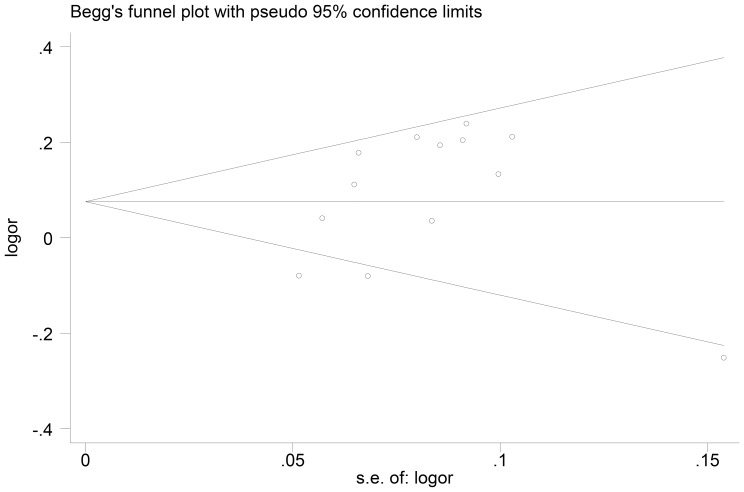
Begg's funnel plot for publication bias (additive model). Each point represents a separate study for the indicated association. Log[or], natural logarithm of the odds ratio. Horizontal line, mean effect size.

Begg's funnel plot and Egger's test were conducted to assess the publication bias of the literatures. As shown in Figure 4, Figure S2 and Figure S3, the shapes of the funnel plots did not reveal any evidence of an obvious asymmetry in any comparison model. Moreover, Egger's test further provided statistical evidence of funnel plot symmetry (*P_Egger's_* = 0.795 for AA vs. GG; *P_Egger's_* = 0.220 for AG vs. GG; *P_Egger's_* = 0.123 for dominant model; *P_Egger's_* = 0.913 for recessive model and *P_Egger's_* = 0.290 for additive model) (Table 4). The results did not indicate any evidence of publication bias.

## Discussion

In this study, we examined the association of the *TERT* rs2736098 polymorphism with the risk for NSCLC in a Chinese Han population. Furthermore, to derive a more precise estimation of the association between this polymorphism and cancer risk, a meta-analysis based on previously published studies and our case-control study was also performed. Our multivariate logistic regression model demonstrated that individuals carrying the A allele or AA genotype exhibited a significantly elevated risk of NSCLC compared with those carrying the G allele or GG genotypes after adjusting for age, gender and smoking status. In the subgroup analysis by histological type, increased cancer risk was observed in adenocarcinomas but not squamous cell carcinomas under the homozygote comparison and the additive genetic model. In addition, the *TERT* rs2736098 variant A allele showed a marginally significant association with overall cancer risk.

The *TERT* rs2736098 polymorphism is mapped to a region of chromosome 5p15.33. The chromosome 5p15.33 locus contains two well-known genes, telomerase reverse transcriptase (*TERT*) and cleft lip and palate trans-membrane 1-like (*CLPTM1L*), which have been implicated in carcinogenesis. Telomerase is expressed in most tumors from virtually all types of cancers, including those of the lung. Telomerase is a relatively specific cancer target, as normal body cells express little or no telomerase for most of their lifespan [11]. Telomere dysfunction in tumor initiation accounts for many aspects of chromosomal instability in human cancers [35]. Cancer cells have been shown to depend on two telomere maintenance mechanisms to gain unlimited proliferation capacity. Generally, telomerase activity is the main mechanism for telomere maintenance. However, 10%–20% of human tumors activate alternative mechanisms of telomere lengthening [36]. The gain at chromosomal region 5p15.33, containing *TERT*, is one of the most frequent genetic events in early stages of non-small-cell lung cancer [37]. Moreover, it has been reported that telomere length may be associated with the risk of lung cancer [38]–[40]. Little is known about the underlying biological mechanism or functional significance of this polymorphism. Although rs2736098 is a synonymous polymorphism, this *TERT* SNP has been shown to be associated with telomere length [15].

Many studies have investigated the role of this polymorphism in the etiology of cancer of various organs, including the bladder, liver, and breast, among others. However, the results of related published case-control studies remain inconsistent [16]–[27].For example, Zhang et al. [24] found that the rs2736098 [A] allele contributed significantly to HCC risk. However, Ding et al. [20] detected no association between the *TERT* rs2736098 polymorphism at 5p15.33 and the risk of HCC. In two population-based case-control studies conducted separately among non-Hispanic whites (NHW) and Asian populations, the *TERT* rs2736098 polymorphism exhibited a significant association with bladder cancer risk among non-Hispanic whites. However, an association of similar magnitude was not observed in the Asian population [21]. In a Polish study of 1,995 breast cancer cases and 2,296 controls, Savage et al. [17] found no evidence that the *TERT* rs2736098 polymorphism at 5p15.33 was associated with breast cancer risk. However, in stratified analysis, this variant exhibited evidence of being associated with a reduced risk of breast cancer among individuals with a family history of breast cancer. Although it is difficult to explain the controversial results in these studies, different genetic backgrounds, cancer types and study designs may contribute to the discrepancies. Interestingly, our case-control study demonstrated that the AA homozygote in *TERT* rs2736098 exhibited a significantly increased risk of developing NSCLC (OR = 1.48, 95% CI = 1.05–2.09, *P* = 0.025), especially adenocarcinoma (OR = 1.67, 95% CI = 1.12–2.50, *P* = 0.013), compared with those who carry the GG genotype. The homozygous AA alleles may be correlated with increased lung adenocarcinoma susceptibility. The results of our case-control study support 5p15.33 (*TERT-CLPTM1L*) as a susceptibility region for lung cancer in the Chinese population [41], [42]. More recently, a Chinese female population study of 501 cancer cases and 576 cancer-free controls also found that the variant allele of rs2736098 was significantly associated with an increased risk of lung cancer, especially in lung adenocarcinomas [25]. Although the underlying biological mechanisms remain largely unknown, differential expression of *TERT* has been observed between adenocarcinoma and other histological carcinomas of lung cancer [43]–[45].

In the current meta-analysis, a borderline significant association between this polymorphism and cancer risk was observed in the overall analysis, with obvious between-study heterogeneity. However, when stratified by tumor sites, the subgroups of lung cancer and bladder cancer failed to exhibit heterogeneity, suggesting that different tumor sites might be a potential source of heterogeneity. Similarly, after stratifying by ethnicity, heterogeneity was largely reduced in both Asian and European populations, suggesting that ethnicity could partly explain the heterogeneity. Therefore, it may be presumed that the heterogeneity exists mainly owing to differences of ethnicity and tumor types. Furthermore, in the subgroup analysis by ethnicity, we found that individuals carrying the A allele or AA and AA/AG genotypes of the *TERT* rs2736098 polymorphism were more likely to exhibit an increased cancer risk among Asians but not among Europeans, possibly because of the differences in genetic backgrounds among different populations. Another plausible hypothesis suggests that the *TERT* rs2736098 polymorphism, a synonymous single nucleotide polymorphism, is only a marker SNP of other functional variants in *TERT* or other nearby genes. However, this hypothesis remains to be tested. In addition, different study designs and inadequate adjustments for confounding factors might explain, to some extent, the inconsistent results in different cancer types and different populations. The evaluation of heterogeneity, influence analysis, and publication bias confirmed the reliability of the meta-analysis.

Some limitations should be addressed in interpreting the results of our case-control study and meta-analysis. First, the sample size of our case-control study was relatively small. Therefore, well-designed population-based studies with large sample sizes and detailed exposure information are needed to further confirm our findings. Additionally, the meta-analysis was based on unadjusted estimates. A more precise analysis should be conducted if more detailed individual data are available, which will allow for an adjusted estimate. Further, in the subgroup analysis stratified by cancer type, the number of studies and subjects analyzed was small, and caution should be taken in interpreting these results. It might be difficult to make a concrete conclusion because few studies were included in the subgroups. Despite these limitations, our meta-analysis also had some advantages. First, significant data were extracted from the related published case-control studies. Second, all studies included in this meta-analysis were case-control investigations and contained available genotype frequencies, which met our inclusion criteria very well.

In conclusion, we found that the *TERT* rs2736098 polymorphism identified in the 5p15.33 region in Caucasians may also predispose to lung cancer, especially adenocarcinomas, in the Chinese population. Moreover, meta-analysis by tumor type suggested that this genetic variant may modify individual susceptibility to lung and bladder cancer. Further studies are required to validate these findings and explain the inconsistent results in different ethnicities and cancer types.

## Supporting Information

Checklist S1 **PRISMA checklist.**
(DOC)

Figure S1 **Flow diagram of the study selection procedure.**
(TIF)

Figure S2 **Begg's funnel plot for publication bias (AA vs. GG).**
(TIF)

Figure S3 **Begg's funnel plot for publication bias (recessive model).**
(TIF)
