# Supplementary figures and images for: Association between the Telomerase Reverse Transcriptase (TERT) rs2736098 Polymorphism and Cancer Risk: Evidence from a Case-Control Study of Non-Small-Cell Lung Cancer and a Meta-Analysis
Source: PLoS One. 2013 Nov 19;8(11):e76372. doi: 10.1371/journal.pone.0076372 (PMC3834105; doi:10.1371/journal.pone.0076372)

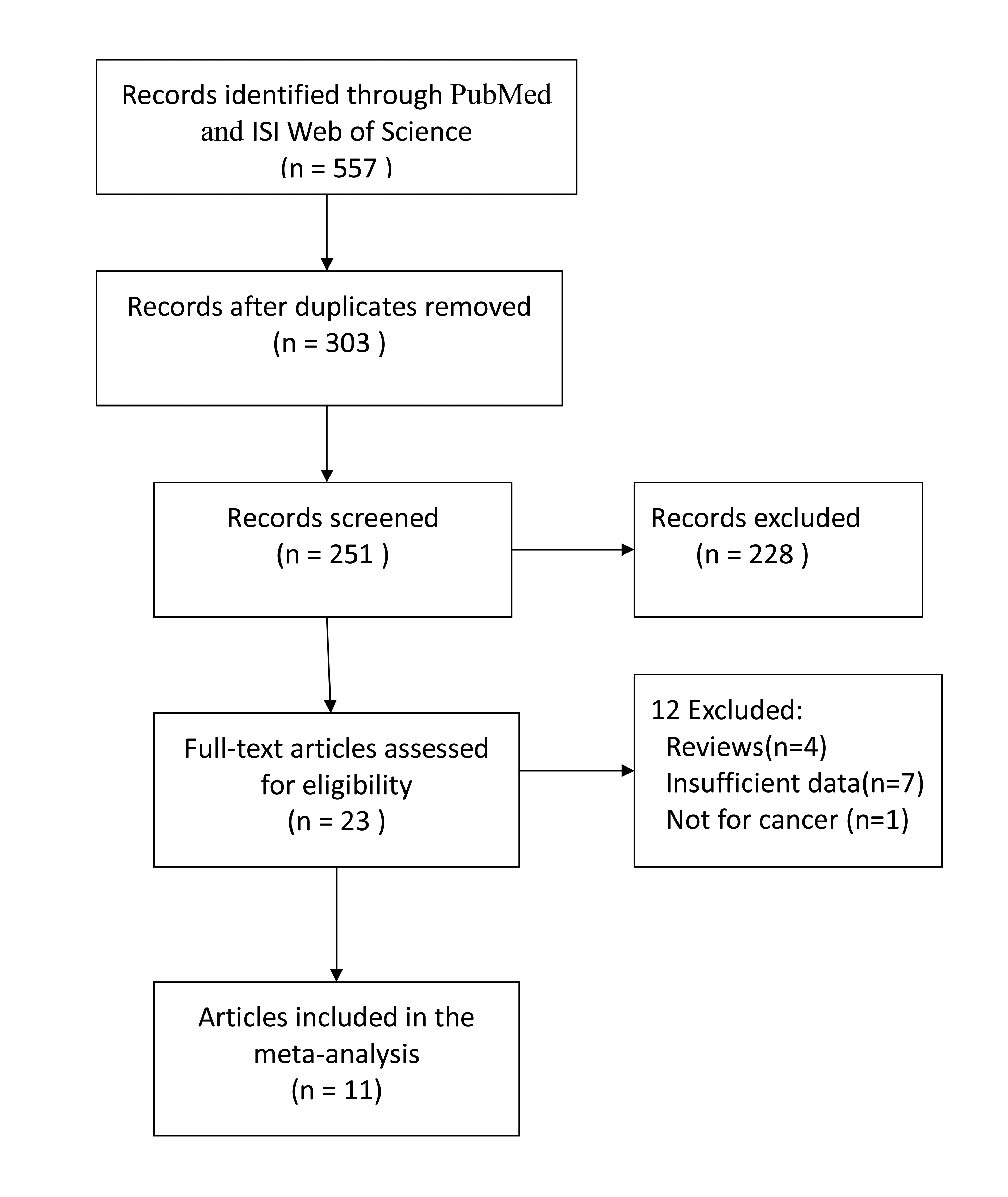

Supplement: Figure S1 — Flow diagram of the study selection procedure. (TIF) [file pone.0076372.s002.tif]

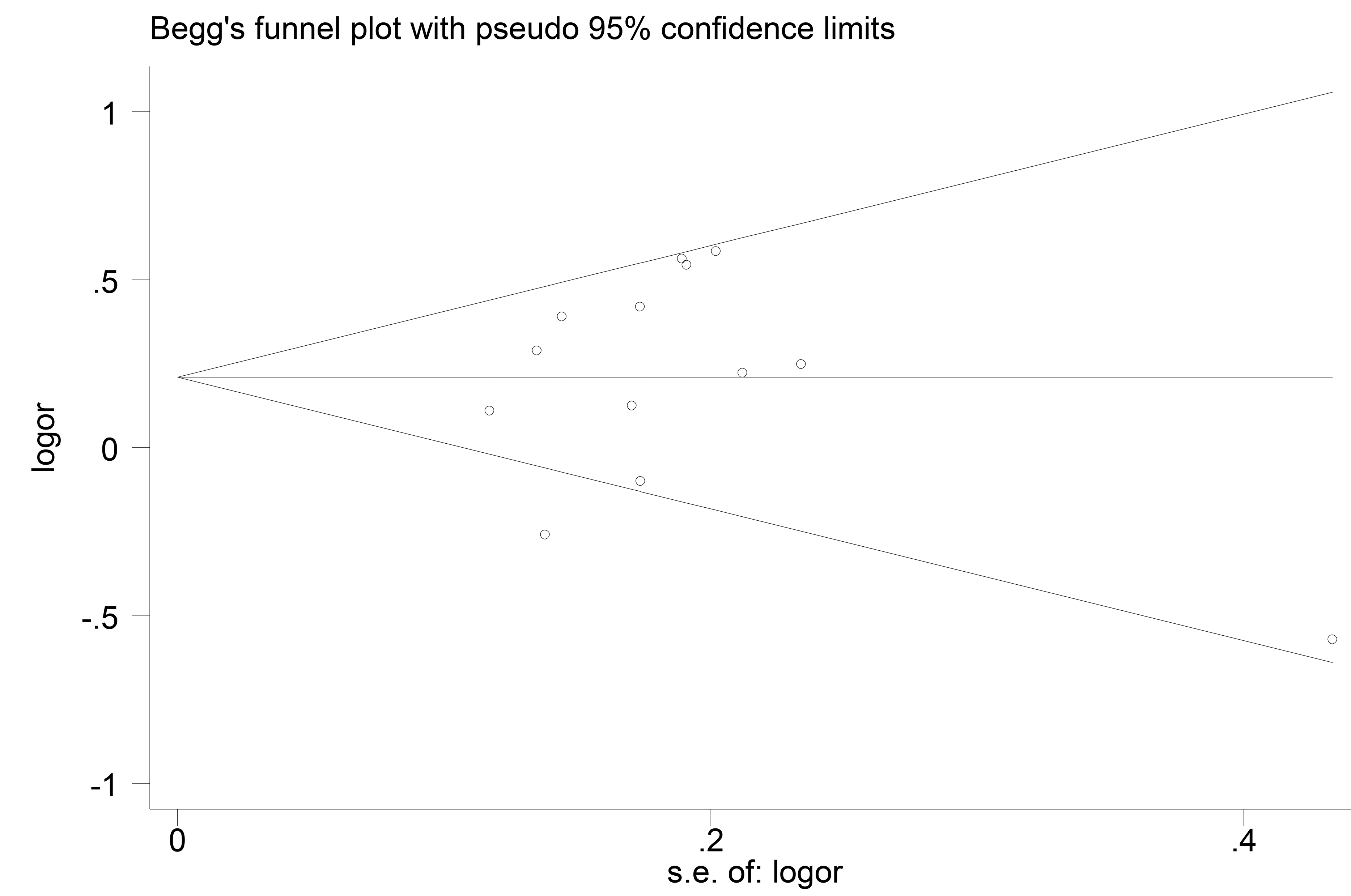

Supplement: Figure S2 — Begg's funnel plot for publication bias (AA vs. GG). (TIF) [file pone.0076372.s003.tif]

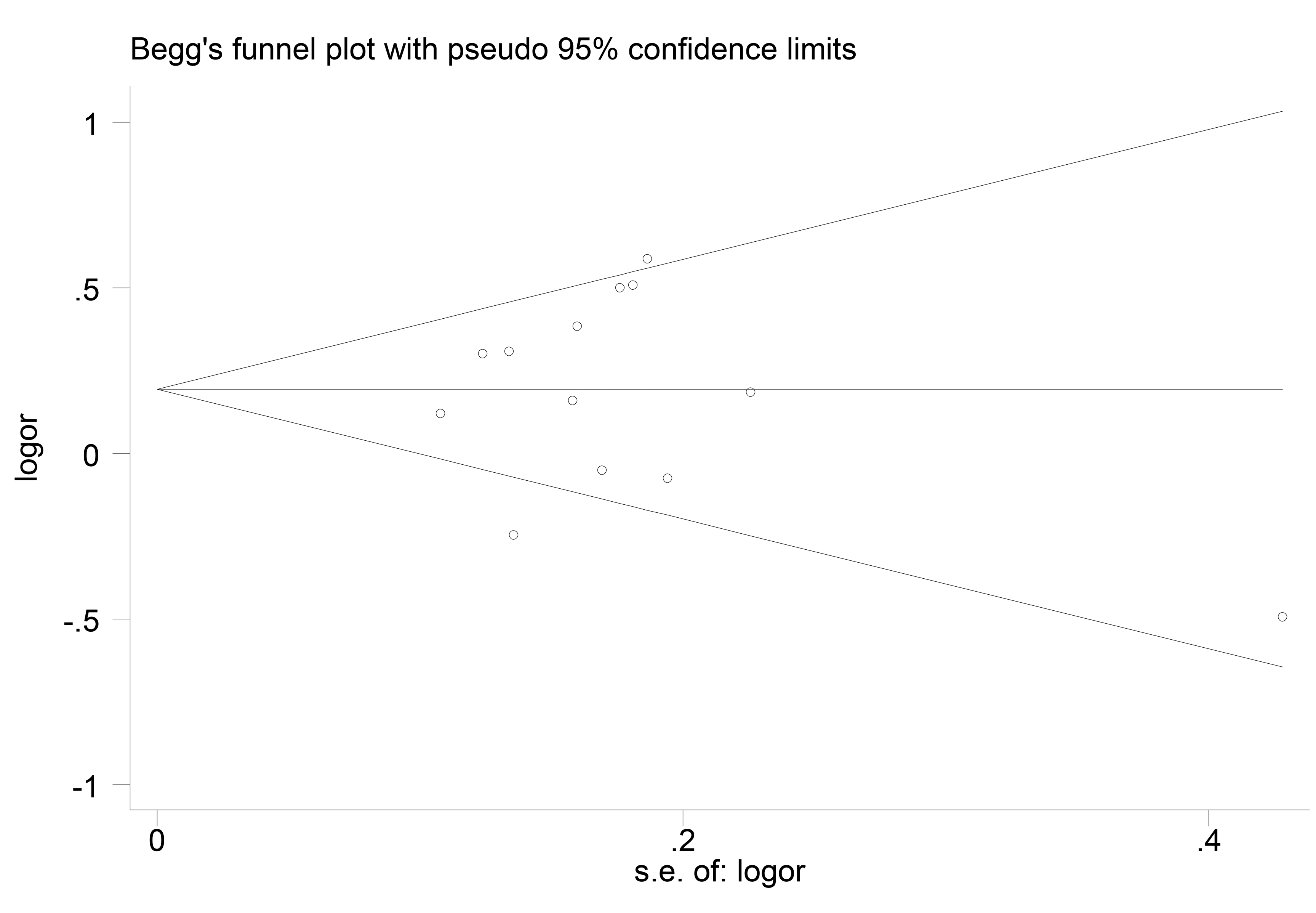

Supplement: Figure S3 — Begg's funnel plot for publication bias (recessive model). (TIF) [file pone.0076372.s004.tif]
